# Consequences of hypoxia-reoxygenation phenomena in patients with obstructive sleep apnea syndrome

**DOI:** 10.4103/0256-4947.75772

**Published:** 2011

**Authors:** Tansu Ulukavak Ciftci, Oguz Kokturk, Senay Demirtas, Özlem Gülbahar, Neslihan Bukan

**Affiliations:** a From the Sleep Disorders Center, Gazi University Faculty of Medicine, Ankara, Turkey; b From the Department of Biochemistry, Faculty of Medicine, Gazi University, Ankara, Turkey

## Abstract

**BACKGROUND AND OBJECTIVES:**

Obstructive sleep apnea syndrome (OSAS) is a common disorder characterized by numerous episodes of absence of respiratory flow during sleep, which can be followed by a decrease in SaO_2_, which is rapidly normalized when ventilation resumes. We hypothesize that this hypoxia–reoxygenation phenomena may affect the generation of vascular endothelial growth factor (VEGF), erythropoietin (EPO), endothelin-1 (ENDO-1), and inducible nitric oxide synthase (iNOS).

**DESIGN AND SETTING:**

Prospective, patients referred to sleep disorders center.

**PATIENTS AND METHODS:**

The presence and severity of OSAS were determined using the standard overnight polysomnography. Diagnosis of OSAS was made when the apnea-hypopnea index (AHI) was ≥15, independent of the appearance of symptoms. Serum levels of VEGF, EPO, ENDO-1, and nitrite–nitrate were measured after overnight fasting in 69 patients with OSAS and in 17 healthy control subjects. Serum levels of VEGF and nitrite–nitrate were measured again after 12 weeks of treatment with continuous positive airway pressure (CPAP) in OSAS patients.

**RESULTS:**

Serum VEGF levels were found to be significantly higher and nitrite-nitrate levels were found to be significantly lower in OSAS patients than in controls (*P*=.003, .008, respectively), but no differences in EPO and ENDO-1 levels were found between the groups. We demonstrated that in OSAS patients, the serum VEGF levels were decreased and nitrate levels were increased after 12 weeks of CPAP treatment (*P*=.001, .002, respectively).

**CONCLUSION:**

According to our data, it is likely that hypoxia–reoxygenation phenomena affect the VEGF and nitrite–nitrate levels, which may be pathogenic factors in generating cardiovascular complications in OSAS.

Obstructive sleep apnea syndrome (OSAS) is characterized by repeated episodes of upper airway obstruction during sleep, associated with increasing respiratory efforts, intermittent arterial oxygen desaturation, systemic and pulmonary arterial blood pressure surges, and sleep disruption. OSAS is highly prevalent disease in the population, affecting ≥4% of males and 2% of females.^1^ The typical OSAS patient is male, middle-aged, and obese. Obesity unfavorably affects respiratory function and may promote the collapse of the upper airway during sleep;^2^ in addition, when body weight increases, the frequency of respiratory events during sleep also increases.^3^

According to recent studies, untreated moderate-to-severe OSAS was associated with increased rates of nonfatal cardiovascular events after a relatively short follow-up.^4^,^5^ At least four longitudinal studies have confirmed increased cardiovascular morbidity in OSAS patients.^6^,^7^ Besides decreasing lung function, obesity increases cardiovascular risk, making it hard to assess the independent role of OSAS on cardiovascular morbidity and mortality. The interrelationships between obesity and OSAS are complex and possibly bidirectional,^8^ and some authors believe that OSAS should be considered as one manifestation of the metabolic syndrome.^9^ The basic mechanisms involved in the increased cardiovascular risk of OSAS remain unclear. In fact, several studies support the existence of oxidative stress and endothelial dysfunction in OSAS, and several authors have suggested a possible involvement of the latter in the pathogenesis of cardiovascular disease in these patients.

OSAS is characterized by the occurrence of numerous episodes of apneas during sleep, which can be followed by a decrease in SaO_2_ that is rapidly normalized when ventilation resumes. We hypothesize that these hypoxia–reoxygenation phenomena may effect the generation of vascular endothelial growth factor (VEGF), erythropoietin (EPO), endothelin-1 (ENDO-1), and inducible nitric oxide synthase (iNOS). To date, no study has evaluated more than two parameters related to oxidative stress in OSAS patients comparing to a control group of similar age and body mass index.

## PATIENTS AND METHODS

We enrolled newly diagnosed cases with OSAS and age- and body mass index-matched control subjects. Subjects were recruited from patients referred to the Sleep Disorders Center of Gazi University Faculty of Medicine for suspected sleep apnea. The study was approved by the Institutional Ethics Committee, and conducted in accordance with the guidelines of the Declaration of Helsinki. The subjects were examined with polysomnography (PSG) and classified as controls according to data of the apnea-hypopnea index (AHI). All patients with OSAS were also diagnosed with PSG. Before enrollment, all subjects gave written informed consent to participation.

Overnight PSG was performed in all patients by a computerized system (Somnostar alpha; Sensormedics, USA) and included the following variables: electrooculogram (2 channels), electroencephalogram (4 channels), electromyogram of submental muscles (2 channels), electromyogram of the anterior tibialis muscle of both legs (2 channels); electrocardiogram, and airflow (with a nasal cannula). Chest and abdominal efforts (2 channels) were recorded using inductive plethysmography, arterial oxyhemoglobin saturation (SaO_2_: 1 channel) by pulse oximetry with a finger probe. The recordings were conducted at a paper speed of 10 mm/s, and sleep stage was scored according to the standard criteria of Rechtschaffen and Kales.^10^ Arousals were scored according to accepted definitions.^11^ The AHI was obtained by dividing the total number of apneas and hypopneas by the total sleep time. Apneas were defined as complete cessation of airflow ≥10 seconds. Hypopneas were defined as a reduction of >50% in one of three respiratory signals, airflow signal or either respiratory or abdominal signals of respiratory inductance plethysmography, with an associated decrease of ≥3 % in oxygen saturation or an arousal.^12^ According to the recently updated International Classification of Sleep Disorders published by the American Academy of Sleep Medicine, a diagnosis of OSAS was made if the AHI is ≥15, independent of occurrence of symptoms, or whenever an AHI >5 is associated with any of the following: (1) sleep attacks or excessive daytime sleepiness, unrefreshing sleep, fatigue or (2) insomnia, or (3) witnessed heavy snoring and/or breathing pauses referred by the partner.^13^ In the control subjects, OSAS was excluded by a negative history of sleep-related symptoms (snoring, witnessed apneas, and excessive daytime sleepiness) and with an AHI <5 at overnight PSG. Patients with sleep disorders, except OSAS, such as upper airway resistance syndrome, periodic leg movement syndrome or narcolepsy, were excluded.

During the first night, a diagnostic study was performed. After confirming the diagnosis of OSAS, continuous positive airwave pressure (CPAP) therapy was given to all patients during the second night (REM Star; Respironics, Murrysville, PA, USA) during 12 weeks. Compliance with treatment was checked by the timer built up in the CPAP device. A priori, good compliance for post-treatment evaluation was defined as the mean usage of CPAP > 4 hours per night for 5 days of the week. According to these criteria, 20 patients were excluded from the follow-up analysis. Diurnal SaO_2_ (SaO_2_ wake), minimum nocturnal SaO_2_ (SaO_2_ min), mean nocturnal SaO_2_ (SaO_2_ mean), and time spent (percent of the recording time) with SaO_2_ less than 90% (desaturation percent) were recorded for all cases. An SaO_2_ value <90% was defined as hypoxia.

Serum levels of VEGF, EPO, ENDO-1, and nitrite-nitrate were measured after overnight fasting. Serum VEGF levels were measured with a solid phase sandwich enzyme linked-immunosorbent assay (ELISA) using a human VEGF kit (Biosource International, Inc., USA). The minimum detectable dose of VEGF is 5 pg/mL with this assay protocol.^14^ Endothelin-1 levels were determined using immunometric EIA (enzyme immunometric assay) kit (Cayman Chemical, Michigan, USA) that permits endothelin measurements within the range of 0-250 pg/mL, typically with a limit a detection of 1.5 pg/mL. inter- and intra-assay CVs are <10%.^15^

Nitric oxide (NO) gas has a short plasma half-life of only a few seconds, and is difficult to measure. More feasible is the measurement of the more stable derivatives of NO (nitrite and nitrate) in biological fluids. Nitrite and nitrate concentrations not only depend on endothelial NO formation, but also other aspects of NO metabolism such as distribution in various body compartments and the rate of elimination by the kidneys. Nevertheless, nitrite and nitrate levels have been suggested as the most appropriate measure of overall NO production.^16^ Nitrate and nitrite levels were determined using nitrate/nitrite colorimetric assay kit (Cayman Chemical, Michigan, USA). The first step was the conversion of nitrate to nitrite using nitrate reductase. In the second step, nitrite concentrations were measured by the Griess reaction.^17^

Means and standard errors of measurement (SEM) were determined for continuous variables and percentage for categorical variables. The significance of differences between the two groups was analyzed with the Mann–Whitney U-test. The correlation was analyzed with the Pearson correlation coefficient. All statistical analyses were carried out using statistical software (SPSS, version 11.0 for Windows; SPSS Inc; Chicago, IL). Differences were considered significant at *P*<.05.

## RESULTS

After 186 subjects underwent PSG, 89 were considered to have OSAS (AHI >15). Twenty patients were excluded from the follow-up analysis because of poor compliance. Sixty-nine patients were included in the OSAS group. Seventeen subjects without OSAS (AHI <5) were enrolled as control subjects. **Table 1** shows the main clinical characteristics of patients with OSAS and healthy controls. **Table 2** shows the values of the different biological markers. Serum VEGF levels were significantly higher and nitrite–nitrate levels were significantly lower in OSAS patients than in controls (*P*=.003, P=.008, respectively), but no differences in EP and ENDO-1 levels were found between the groups. In OSAS patients, serum VEGF levels were decreased and nitrate levels were increased after 12 weeks of CPAP treatment (*P*=.001, *P*=.002, respectively) (**Table 3**). VEGF levels were significantly and negatively correlated with SaO_2_ (wake), SaO_2_ (min), SaO_2_ (mean), and desaturation percent in all cases (n = 186) (*P*=.01, *P*=.001, *P*=.003, *P*=.04, respectively) and in OSAS patients (n=69) (*P*=.03, *P*=.001, *P*=.03, *P*=.03, respectively). There was no significant correlation between other parameters and SaO_2_ both in OSAS patients and all cases.

**Table 1:** The main clinical characteristics of patients with OSAS and healthy controls.

	OSAS Patients	Control Group	*P*
Sex (M/F)	49/20	11/6	
Age (mean ± SD)	53.27 (11.38)	51.5 (10.5)	.58
Body mass index	30.9 (6.2)	29.1 (4.6)	.33
Apnea-hyponea index	48.4 (30.4)	1.89 (1.1)	<.001
SaO_2_ (wake)	94.5 (1.8)	95.3 (1.5)	.17
SaO_2_ (min)	73.9 (9.5)	87.6 (3.1)	<.001
SaO_2_ (mean)	88.3 (3.9)	92.6 (2.1)	<.001
Desaturation percent	38.02 (35.67)	12.75 (26.8)	<.001

Diurnal SaO_2_ (SaO_2_ wake), minimum nocturnal SaO_2_ (SaO_2_ min), mean nocturnal SaO_2_ (SaO_2_ mean), and time spent (percent of the recording time) with SaO_2_ <90% (desaturation percent).

**Table 2:** Serum VEGF, nitrite-nitrate, EPO, and ENDO-1 levels in OSAS patients and in control group.

	OSAS Group	Control Group	*P*
VEGF	168.16 (3.7)	89.1 (11.4)	.003
Nitrite-nitrate	0.5 (0.5)	0.81 (0.7)	.008
EPO	10.8 (6.0)	9.7 (5.1)	.5
ENDO-1	36.7 (5.4)	37.7 (5.4)	.2

VEGF: vascular endothelial growth factor, EPO: erythropoietin, ENDO-1: endothelin-1.

**Table 3:** Serum VEGF and nitrite-nitrate levels in OSAS patients at date of diagnosis and after 12 weeks of CPAP treatment.

	Before CPAP	After CPAP	*P*
VEGF	168.16 (3.7)	106.05 (8.5)	.001
Nitrite-nitrate	0.5 (0.5)	0.86 (0.4)	.002

VEGF: vascular endothelial growth factor.

## DISCUSSION

Reperfusion–reoxygenation injury refers to the damage that occurs as a result of restoration of the blood circulation to an ischemic or hypoxic tissue. Although several mechanisms inflict this damage, it is mainly attributed to free radical production during reoxygenation. Hypoxia/reoxygenation induces complex metabolic and molecular changes including: (1) changes in energy metabolism, (2) alterations in gene expression, and (3) induction of cell surface molecules. All are intercalated with altered free radical flux, which affects NO bioavailability as well.

NO, an intracellular signaling molecule as well as a free radical, is affected by hypoxia. NO is the main vasodilating substance released from the endothelium. Deficiency of NO has been implicated in the pathogenesis of cardiovascular disease.^18^,^19^ In our study, plasma levels of nitrate were found to be markedly reduced in a group of 69 untreated patients with OSAS. After 12 weeks of CPAP therapy, nitrate levels increased in this group. Ip and colleagues reported suppression of circulating NO derivatives in OSAS, rapidly reversible upon onset of nasal continuous positive airway pressure (nCPAP) therapy.^20^ Ip and associates failed to mention some possibilities. First, as oxygen is a cosubstrate of NO synthase (NOS), OSA-related nocturnal desaturation might result in depressed synthesis of NO. Second, nighttime hypoxia might suppress the transcription of the endothelial NOS gene and the stability of its mRNA as suggested by cell culture experiments performed under hypoxic conditions. Third, it has been demonstrated that NOS inhibitors are elevated in OSAS and thus, might also contribute to lowered nitrite/nitrate levels. Finally, it is quite possible that NO is scavenged by free oxygen radicals generated under conditions of hypoxia–reoxygenation by circulating neutrophils.^20^ Ohike et al’s findings suggest that nCPAP reduces the concentration of asymmetric NG–-NG-dimethylarginine (ADMA), which is an endogenous inhibitor of endothelial NO synthesis, thereby enhancing NO production and leading to an improvement of endothelium-dependent vasodilatation.^21^ Hypoxia induces the activation of the adaptive pathway mediated by upregulation of the transcription factor hypoxia-inducible factor (HIF-1). There is evidence of HIF-1 dependent gene activation in OSAS as indicated by increased levels of VEGF.

VEGF is a vasodilator that induces the proliferation of endothelial cells, increases vascular permeability, and causes greater production of NO and prostacyclin. Hypoxia is believed to stimulate the production of VEGF. Investigators have sought to implicate VEGF as part of the mechanism by which OSAS results in greater incidence of cardiovascular disease.^22^ Our results showed that the concentrations of VEGF were significantly higher in OSAS patients than in healthy controls and were decreased after 12 weeks of CPAP treatment. Many, but not all studies, have found elevated VEGF levels in subjects with OSAS. In study of Phillips et al, 1 week of CPAP withdrawal was associated with a return of OSAS and marked increase in sympathetic activity with out a concomitant elevation of VEGF.^23^ In contrast, VEGF levels were found elevated in OSAS in some studies.^24^ Apnea-associated hypoxia is also likely to play a significant role in the increased levels of VEGF found in OSAS, because levels have been found to rapidly decrease after one night of oxygen administration.^25^ According to the importance of hypoxia for VEGF levels, our study showed that the mean value of time spent with SaO_2_ less than 90% (desaturation percent) was higher in OSAS patients than that of controls, VEGF levels were significantly and negatively correlated with SaO_2_ (wake), SaO_2_ (min), SaO_2_ (mean), and desaturation percent.

Erythropoietin regulates the red blood cell mass. Sustained cellular hypoxia is associated with the activation of a ubiquitous transcriptionally initiated response mediated by the transcription factor HIF-1. HIF-1 is activated in sustained hypoxia through a well-defined mechanism, resulting in increased expression of a number of genes encoding proteins such as EPO. Previous reports have indicated that there is activation of the HIF-1 pathway in OSAS. In our study, there was no difference in EPO levels between the groups. EPO levels have been reported as increased in OSAS, but this increase was observed only in patients with severe disease and a higher body mass index.^26^ A possible explanation for these different findings is that patients with severe OSAS may be exposed to a sufficient cumulative period of sustained hypoxia during sleep to activate the HIF-1-dependent pathway.^27^

Endothelin-1 (ET-1) is a potent vasoconstrictive and mitogenic peptide. Recently, evidence has emerged suggesting a role for an enhancer of chemoreceptor activity, endothelin (ET), as a mediator of acclimatization. ET is a 21-amino acid peptide found in endothelium and in type 1 cells (glomus cells) in the carotid bodies.^28^ Chen and colleagues have presented evidence, based on reverse transcriptase polymerase chain reaction, that continuous hypoxia increases expression of the ET receptor and of preproendothelin. An association between ENDO-1 and OSAS could be assumed but in our study there was no difference in ET levels between the groups. Similarly, Diefenbach and colleagues showed that ET plasma levels of untreated OSAS patients and of patients treated with nasal CPAP were not elevated compared with controls.^29^ Conversely, Gjorup et al studied the OSAS patients and control subjects during the nighttime with serial determinations of ET in plasma and showed that OSAS patients had a higher plasma level of ET than healthy controls and the mean nocturnal level of ET correlated significantly to the AHI.^30^ According to our data, it is likely that hypoxia-reoxygenation phenomena affect the VEGF and nitrite-nitrate levels, which may be pathogenic factors in generating cardiovascular complications in OSAS.
